# Oral Delivery of Niclosamide as an Amorphous Solid Dispersion That Generates Amorphous Nanoparticles during Dissolution

**DOI:** 10.3390/pharmaceutics14122568

**Published:** 2022-11-23

**Authors:** Miguel O. Jara, Zachary N. Warnken, Sawittree Sahakijpijarn, Rishi Thakkar, Vineet R. Kulkarni, Dale J. Christensen, John J. Koleng, Robert O. Williams

**Affiliations:** 1 Molecular Pharmaceutics and Drug Delivery Division, College of Pharmacy, The University of Texas at Austin, 2409 University Avenue, Austin, TX 78712, USA; 2 Via Therapeutics, Austin, TX 78712, USA; 3 TFF Pharmaceuticals, Inc., Fort Worth, TX 76107, USA

**Keywords:** niclosamide, amorphous solid dispersion, enteric capsules, enteric-coated tablet, amorphous nanoparticles, hot-melt extrusion, liquid–liquid phase separation, biorelevant media

## Abstract

Niclosamide is an FDA-approved anthelmintic that is being studied in clinical trials as a chemotherapeutic and broad-spectrum antiviral. Additionally, several other applications are currently in the preclinical stage. Unfortunately, niclosamide is a poorly water soluble molecule, with reduced oral bioavailability, which hinders its use for new indications. Moreover, niclosamide is a poor glass former; in other words, the molecule has a high tendency to recrystallize, and it is virtually impossible to generate a stable amorphous solid employing the neat molecule. Previously, our group reported the development of an amorphous solid dispersion (ASD) of niclosamide (niclosamide ASD) that generates nanoparticles during its dissolution, not only increasing niclosamide’s apparent solubility from 6.6 ± 0.4 to 481.7 ± 22.2 µg/mL in fasted state simulated intestinal fluid (FaSSIF) but also its oral bioavailability 2.6-fold in Sprague–Dawley rats after being administered as a suspension. Nevertheless, niclosamide ASD undergoes recrystallization in acidic media, and an enteric oral dosage form is needed for its translation into the clinic. In this work, we further characterized the nanoparticles that generated during the dissolution of the niclosamide ASD. Cryogenic transmission electron microscopy (Cryo-TEM) and wide-angle X-ray scattering (WAXS) revealed that the nanoparticles were amorphous and had a particle size of ~150 nm. The oral dosage forms of niclosamide ASD were formulated using commercial enteric capsules (Capsuline^®^ and Eudracap^TM^) and as enteric-coated tablets. The enteric dosage forms were tested using pH-shift dissolution and acid-uptake tests, using the USP type II dissolution apparatus and the disintegration apparatus, respectively. The capsules exhibited a higher percentage of weight gain, and visual rupture of the Capsuline capsules was observed. Eudracap capsules protected the formulation from the acidic media, but polymer gelling and the formation of a nondispersible plug were noted during dissolution testing. In contrast, enteric-coated tablets protected the formulation from acid ingress and maintained the performance of niclosamide ASD granules during dissolution in FaSSIF media. These enteric-coated tablets were administered to beagle dogs at a niclosamide dose of 75 mg/kg, resulting in plasma concentrations of niclosamide higher than those reported in the literature using solubilized niclosamide at a higher dose (i.e., 100 mg/kg). In summary, an enteric oral dosage form of niclosamide ASD was formulated without hindering the generation of nanoparticles while maintaining the increase in the niclosamide’s apparent solubility. The enteric-coated tablets successfully increased the niclosamide plasma levels in dogs when compared to a niclosamide solution prepared using organic solvents.

## 1. Introduction

Over the years, newly discovered active pharmaceutical ingredients (API) in pharmaceutical pipelines have become increasingly larger in terms of molecular weight, more lipophilic, and much less water soluble and bioavailable [1,2,3]. In 2010, Loftsson and Brewster (2010) reported that approximately 40% of marketed drug products and 90% of drug candidates in development were poorly water soluble [4]. We were unable to identify a more current reference reflecting a more recent contemporary pipeline. Several formulation strategies can be used to increase the aqueous solubility and rate of dissolution of an API including particle size reduction (e.g., mechanical milling by micronization or nanonization), cocrystals, polymorphism, salts, amorphization, amorphous solid dispersions (ASD), cyclodextrins, self-emulsifying drug delivery systems, cosolvents, surfactants, and lipid-based formulations [5,6]. Among these different formulation strategies available to increase the water solubility of APIs, ASDs are attractive, as exemplified by the increasing number of publications and FDA-approved drug products based on this approach [5,7]. In the drug delivery field, ASD often refers to a glassy solution, in other words, a metastable system comprising an amorphous drug–polymer composite. The API is solubilized by the amorphous polymeric matrix [8]. These glassy materials are thermodynamically more energetic than the crystalline ones, leading to increased solubility and dissolution rates [9]. High ASD dissolution is achieved when polymer and API dissolve simultaneously; this occurs at a range of drug loadings, and the maximal drug loading is known as the limit of congruency (LoC) [10]. It has been reported that some ASDs can generate nanoparticles during their dissolution [11]. These nanoparticles act as a reservoir and replenish drug concentrations during the dissolution and diffusion processes of the ASD [12]. This phenomenon is referred to as liquid–liquid phase separation (LLPS) or glass–liquid phase separation (GLPS), where a drug-rich phase is formed when the drug concentration exceeds its amorphous solubility [10,13]. The mechanism for the formation of these nanoparticles is not entirely understood, but there is a dependence on polymer–drug interactions. Additionally, a drug’s glass transition temperature (Tg) and hydrogen bond donor groups are likely relevant [14]. Additionally, incorporating surfactants in the ASD composition seems relevant to further increasing nanoparticle generation during the dissolution process, and the surfactant–drug ratio is an important parameter for improving the oral bioavailability [15]. However, the generation of drug nanoparticles is surfactant specific; it depends on the specific intermolecular interactions between the API and polymer, affecting the particle size and their colloidal stability [16]. Liu et al. (2022) demonstrated that water-resistant drug–polymer interactions form these nanoparticles; in other words, drug–polymer interactions are maintained during dissolution without being disrupted by water. When ionizable moieties are involved, these interactions can depend on the pH of the dissolution media [17]. It has been noted that most ASD-related publications focus on more fundamental aspects of polymer–drug selection [18,19]. Even though these are important considerations when formulating an ASD, they may not be relevant if applied to ASDs that are not properly formulated in a final dosage form.

Interestingly, the literature related to the development and troubleshooting of solid oral dosage forms containing ASDs is limited. It has been described that encapsulated ASDs tend to form a nondispersible plug, hindering drug release [20]. This phenomenon also occurs during the dissolution of tablets containing ASDs [21,22]. This poor dissolution performance is related to polymer gelling, which impedes the disintegration of the oral dosage form, causing drug crystallization [23]. Additionally, the optimum balance between drug release and friability is quite challenging to achieve when preparing tablets containing ASDs [21].

Recently, our group reported a ternary ASD of niclosamide (niclosamide ASD), prepared by hot-melt extrusion (HME), that improved the apparent solubility of niclosamide by more than 60-fold, mainly due to the generation of drug nanoparticles. Niclosamide is a poorly water soluble molecule that was approved by the FDA, in 1982, as an anthelmintic. Recently, niclosamide has been reported in several drug repurposing studies as a chemotherapeutic, antibacterial, and broad-spectrum antiviral (including SARS-CoV-2), among other indications, as described in several published review papers [24,25]. The niclosamide ASD formulation increased niclosamide’s oral bioavailability in rats. However, niclosamide in the ASD undergoes recrystallization if exposed to acidic media [26]. Consequently, the oral administration of niclosamide ASD would be inefficient without being included in a proper enteric dosage form. This can be an applicable to other ASDs composed of weakly acidic APIs and/or polymers. As an example, using a weakly acidic polymer, Elkhabaz et al. (2019) observed a drastic drop in the supersaturation when running pH-shift tests using an ASD composed of posaconazole (weakly basic API) and HPMC-AS (weakly acidic polymer). During the acid phase, the polymer could not protect the drug from recrystallization at higher drug loadings, leading to a poor dissolution performance of the ASD [27].

In this work, we aimed (i) to further understand the solid-state properties of the nanoparticles that were generated during dissolution; (ii) to select a proper enteric (delayed release) dosage form for the delivery of niclosamide ASD; and (iii) to conduct in vivo pharmacokinetic testing in beagle dogs. Our hypothesis was that an enteric-coated dosage form would protect the formulation from recrystallization without hindering the generation of the nanoparticles, therefore increasing the oral bioavailability of niclosamide.

## 2. Methods

### 2.1. Preparation of the Niclosamide ASD Granules

Niclosamide anhydrate was purchased from Shenzhen Nexconn Pharmatechs LTD (Shenzhen, China); Kollidon^®^ VA64 (PVP–VA) and Kolliphor^®^ TPGS (D-ɑ-tocopheryl polyethylene glycol succinate, TPGS) were obtained from BASF Corporation (Tarrytown, NY, USA). Niclosamide, PVP-VA, and TPGS were admixed at a 60:35:5 ratio and extruded using a ZSE 12 HP-PH (Leistritz Advanced Technologies Corp., Nuremberg, Germany). The extrusion was performed at a feed rate of 3 g/min with a screw speed of 50 rpm. The operating temperatures are specified in the Supplemental Information, Table S1. Then, granules of niclosamide ASD were obtained by milling the extrudates using a Fitzmill Comminutor Model L1A Benchtop Mill (The Fitzpatrick Company, Westwood, MA, USA) operating at 5000 rpm and equipped with a 0.06″ round hole screen.

### 2.2. Preparation of the Enteric Capsules Containing Niclosamide ASD Granules

The niclosamide ASD granules were admixed with sodium bicarbonate (Spectrum chemical, New Brunswick, NJ, USA) and sodium starch glycolate (Explotab^®^, JRS Pharma, Patterson, NY, USA) in a 70/25/5 ratio. This mixture was filled into either Capsuline^®^ (HPMCP/HPMC) (Dania Beach, FL, USA) or Eudracap^TM^ (HPMC/Eudragit^®^ polymers) capsules, size 0. The Eudracaps were kindly donated by Evonik Corporation–North America (Piscataway, New Jersey, NJ, USA).

### 2.3. Preparation of the Enteric-Coated Tablets of Niclosamide ASD

The enteric-coated tablets were prepared by first admixing 66.63% niclosamide ASD granules, 13.1% microcrystalline cellulose (Ceolus^®^ PH-102, Asahi Kasei Chemical Corporation, Tokyo, Japan), 8.8% croscarmellose sodium (Kiccolate^TM^, Nichirin Chemical Industries, LTD., Dojima, Japan), 0.28% magnesium stearate (Spectrum Chemical, New Brunswick, NJ, USA), and 11.1% sodium chloride (Fisher Chemical, Ottawa, ON, Canada). The blend was filled into a hopper placed on a Stoke F4 Press. The tablet cores were produced using a 10 mm round standard cup tooling and with a target final tablet core weight of 428 mg. Then, the tablet cores were added to a Vector LDCS-Hi Coater (Vector Corporation, Marion, IA, USA). Opadry II Clear (Colorcon, West Point, PA, USA) dispersion in either water or 20:80 water/ethanol mixture was used for seal coating to a target weight gain of 3%. Then, the sub-coated tablets were enteric coated with either a completely water-based coating (Acryl-EZE^®^ 93 A, Colorcon, West Point, PA, USA) or a mixture of water and ethanol-based coating (Opadry^®^ Enteric, Colorcon, West Point, PA, USA) to a target 10% weight gain.

### 2.4. HPLC Analysis

The samples from the dissolution tests were measured using a Dionex Ultimate 3000 HPLC system (Thermo Fisher Scientific Inc., Sunnyvale, CA, USA) at a 1.5 mL/min flow rate, and the UV detector was set at 331 nm and employing a ZORBAX SB-C18 column (4.6 × 250 mm, 5 µm) (Agilent Technologies, Palo Alto, CA, USA). The mobile phase was composed of (A) 0.3% formic acid in water solution and (B) acetonitrile (Fisher Scientific, Pittsburgh, PA, USA) mixed in a 40:60 ratio.

### 2.5. Dissolution Testing of the Granules, Capsules, and Tablets

The niclosamide ASD granules, capsules, and tablets underwent dissolution tests using a USP type II apparatus (Hanson SR8-Plus apparatus, Hanson Research Co., Chatsworth, CA, USA) with low volume vessels (150 mL) at 37.0 ± 0.5 °C and a paddle speed of 100 rpm. The biorelevant medium FaSSIF (biorelevant.com Ltd., London, UK) was used to simulate the intestinal dissolution medium present in humans. It was prepared according to the instructions from the manufacturer, using a buffer pH 6.5, composed of sodium hydroxide, monobasic sodium phosphate (Fisher Scientific, Fair Lawn, NJ, USA), and sodium chloride (Sigma Aldrich, Saint Louis, MO, USA).

A 230 mg sample of niclosamide ASD granules (equivalent to 80 mg of niclosamide) underwent the dissolution test in 150 mL of FaSSIF. In the case of the oral dosage forms, enteric capsules were placed inside sinkers to maintain them on the bottom of the vessel. Tablets, on the other hand, did not require sinkers. The samples were collected and filtered using a 25 mm 0.2 µm Nylon syringe filter (VWR International LLC, Radnor, PA, USA) for analysis by HPLC. For the pH-shift dissolution test, the dosage forms were first added to 120 mL of 0.1 N HCl for 2 h. Then, the tablets/capsules were transferred to 150 mL of FaSSIF medium. Testing of the Eudracap capsules required the modification of the testing, increasing the FaSSIF buffer pH to 6.8 due to the increased pH required to dissolve the capsule based upon the shell polymer chemistry.

### 2.6. Acid Uptake Test

To determine the acid resistance of the enteric oral dosage forms, six capsules/tablets were weighed and placed into a QC-21 disintegration test system (Hanson Research, Chatsworth, CA, USA) containing 0.1 N HCl at 37 °C for 2 h. Sinkers were used in the case of capsules. After the test, the capsules/tablets were blotted to remove the surface liquid and weighed again to determine the weight increase.

### 2.7. Polarized Light Microscopy (PLM)

The niclosamide ASD granules were placed on a glass slide and observed using an Olympus BX-53 polarized light microscope (Olympus Corporation of the Americas, Center Valley, PA, USA) with a first-order red compensator and 10× and 20× objectives. The pictures were taken using a QImaging QICAM digital camera (QImaging, Surrey, BC, Canada).

### 2.8. Wide-Angle X-ray Scattering (WAXS)

The niclosamide anhydrate was prepared by heating the niclosamide raw material at 100 °C for 15 min [28]. This must be performed due to the hygroscopic nature of niclosamide. Niclosamide Ha (hydrate) was prepared by pouring niclosamide raw material in water and then letting it dry at ambient temperature for 24 h under a hood.

Wide-angle X-ray scattering (WAXS) was employed to determine the crystallinity of the nanoparticles generated during dissolution in FaSSIF. After 2 h of dissolution, 5 mL were collected and added into a Float-A-Lyzer^®^ Device (Repligen, Rancho Dominguez, CA, USA) for dialysis. The sample was dialyzed using 1 L of water supersaturated with niclosamide (added in excess) for 24 h. This was conducted to remove the excess inorganic salts and bile salts from the sample and to increase the measurement resolution. Then, the sample was collected and immediately frozen using liquid nitrogen. Thereafter, the sample was lyophilized using an SP VirTis Advantage Pro shelf lyophilizer (SP Industries, Inc., Warminster, PA, USA) to obtain a dry powder. The primary drying process was at −40 °C for 20 h, and then the temperature was linearly increased to 25 °C over 20 h, followed by holding the temperature at 25 °C for 20 h. The pressure was maintained at 100 mTorr during the lyophilization process. The samples were mounted at the sample position inside the equipment using a sandwich of Scotch^®^ Magic Greener Tape and Scotch^®^ Permanent Double-Sided Tape (3M, Saint Paul, MN, USA), similar to the methodology used by Hövelmann et al. (2019) [29]. The samples were analyzed using a SAXSLab instrument (WAXSLab, Northampton, MA, USA) equipped with a PILATUS3 R 300 K detector (DECTRIS Ltd., Philadelphia, PA, USA). The instrument was operated using the Ganesha instrument software (SAXSLab, Northampton, MA, USA).

In addition, WAXS was used to analyze the potential recrystallization of the encapsulated niclosamide. The samples of the enteric capsules underwent 2 h of dissolution testing in acid medium (0.1 N HCl); then, they were opened and the content was dried for 24 h at ambient temperature under a hood. In the case of the Capsuline sample, the dried material was milled to obtain a powder. The sodium bicarbonate and Explotab^®^ were analyzed as received. The powder samples were added to 2 mm glass capillaries (Hampton Research, Aliso Viejo, CA, USA). Each sample was analyzed using a 2 mm off-centered beam stop mask for an acquisition time of 600 s.

### 2.9. Cryogenic Transmission Electron Microscopy (Cryo-TEM)

The samples of niclosamide ASD were prepared using the same methodology of dialysis described in the WAXS section. These liquid samples were added into a 20 s glow discharged Q2/2R 200 mesh copper grid and vitrified in ethane using a Vitrobot Mark IV (FEI, Millsboro, OR, USA) at 100% relative humidity. The samples were observed using a Glacios Cryo-TEM with Falcon 4 (Thermo Scientific, Waltham, MS, USA). The magnification was 92,000X, and the dose rate was ~2.02e^−^/Å^2^ s. The images were visualized, and their contrast was increased using the ImageJ software, version 1.53t (NIH, Bethesda, MR, USA).

### 2.10. Administration of Niclosamide ASD to Beagle Dogs

In vivo testing in fasted male beagle dogs (8 kg, 7 to 10 months old), following oral administration of enteric-coated tablets containing niclosamide ASD at a dose of 75 mg/kg, was performed. The dog study was conducted under approval from the ITR Laboratories Animal Care and Use Committee. The OLAW animal welfare number is F16-00201 (A5834-0). The animals were kept in an environment with a temperature of 21 ± 3 °C, relative humidity of 50 ± 20%, and 12 h light and 12 h dark. Following each dose administration, the animals were administered 5 mL of water to facilitate swallowing. 1 mL of blood was collected from the animals, and the samples were processed into plasma by centrifugation at 2500 rpm for 10 min at approximately 4 °C. The samples were analyzed by HPLC/MS. The limit of quantification was 40 ng/mL.

## 3. Results and Discussion

### 3.1. Niclosamide ASD Generated Amorphous Nanoparticles in Biorelevant Media

As previously reported, the niclosamide ASD formulation generates nanoparticles during its dissolution in FaSSIF biorelevant media; these particles were observed even after filtering the dissolution test media through 0.2 µm filters [26]. However, initially, their crystallinity was not determined. In theory, these nanoparticles should result from a phenomenon called liquid–liquid phase separation (LLPS) or glass–liquid phase separation (GLPS), where a drug-rich phase is formed when the drug concentration exceeds its amorphous solubility [10,13].

**Figure 1:**
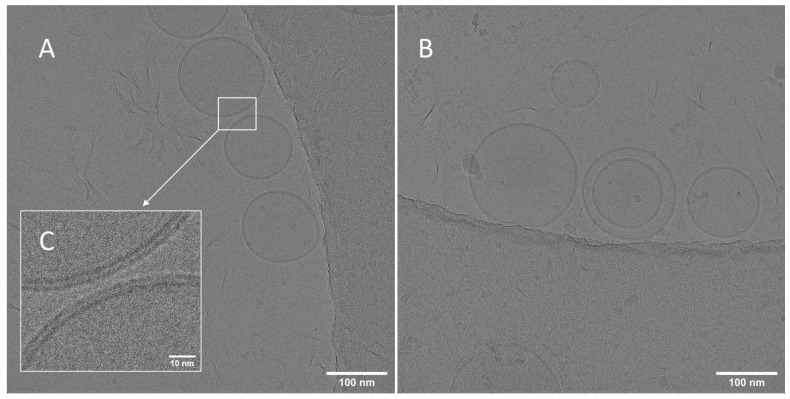
(**A**,**B**) Cryo-TEM of niclosamide ASD nanoparticles after 24 h of dialysis in niclosamide-supersaturated water (5 mL of the sample over 1 L of water) to remove excess inorganic and bile salts. (**C**) Even after the 24 h dialysis, these particles were covered by a bilayer membrane corona of ~4.5 nm. The samples had a niclosamide concentration of 256.60 ± 15.60 µg/mL (measured before vitrification).

Cryo-TEM was conducted to directly observe these drug-rich metastable regions generated by niclosamide ASD in FaSSIF, as shown in Figure 1, as other more common techniques, such as nanoparticle tracking analysis and scanning electron microscopy, were unable to successfully resolve the nanoparticles. Surprisingly, the nanoparticles were spherical, similar to the ones previously reported in the literature [11,12,30]. Furthermore, the particles were surrounded by a bilayer membrane structure of approximately 4.5 nm (Figure 1C). These are in agreement with the bilayer structures generated by FaSSIF that were reported by Clulow et al. (2017). They proved that FaSSIF media forms vesicles of 30–50 nm with bilayer membranes of approximately 5 nm in thickness using cryo-TEM. The bilayer membrane had a similar thickness to that measured for the niclosamide ASD particles [31]. Previously, we reported FaSSIF particles of ~66 nm, measured by dynamic light scattering (DLS) [26]. These are in agreement with the sizes reported by Khoshakhlagh et al. (2015), Kabedev et al. (2021), and Kloefer et al. (2010) of 70, 75, and 30–70 nm nanostructures using DLS, respectively [32,33,34]. These varieties of nanostructures were identified as vesicles, spherical-mixed micelles, disk micelles, and liposomes by cryo-TEM [31,34].

We recently reported that the niclosamide ASD nanoparticles exhibited a mean size of ~100 nm, and cryo-TEM showed particles sizes of approximately ~136 nm [26]. In addition, Harmon et al. (2016) reported interesting behavior of ASDs prepared with increasing concentrations of TPGS. Specifically, increasing concentrations of TPGS facilitated the disintegration of the ASD and led to a greater number of nanoparticles with reduced particle size. TPGS was found on the surface of nanoparticles and acted as a stabilizer [11]. Likewise, in our system, the bile salts in the FaSSIF media plus the TPGS in the ASD could facilitate the formation and stabilization of these nanoparticles. The formation of the nanoparticles did not happen to the same extent without FaSSIF (Supplemental Information, Figure S1). This combination of TPGS and biorelevant media led to an increased limit of congruency and simultaneous release of niclosamide and PVP-VA, as described by Saboo et al. (2021). Interestingly, this effect on the LoC was also observed in prototype formulations using different polymers at different drug loadings of niclosamide, including with other surfactants (Supplemental Information, Figure S2).

**Figure 2:**
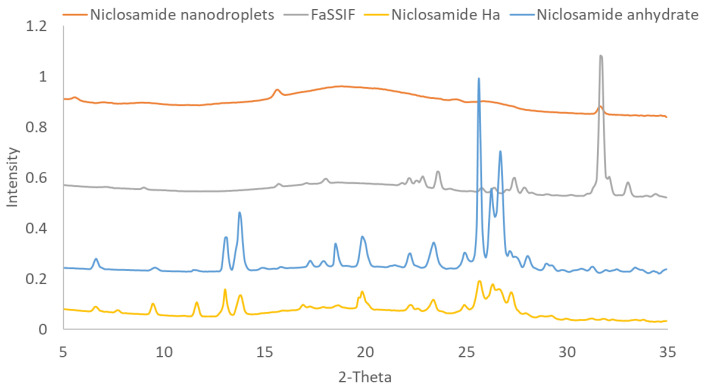
WAXS of niclosamide ASD nanoparticles after 24 h of dialysis in niclosamide-supersaturated water (5 mL of the sample over 1 L of water) to remove excess inorganic and bile salts. Before lyophilization, the samples had a niclosamide concentration of 256.60 ± 15.60 µg/mL (measured before vitrification). Ha: niclosamide hydrate Ha.

After confirmation and elucidation of the nanoparticle morphology by cryo-TEM, further solid-state characterization of the nanoparticles was performed to determine if the particles were amorphous resulting from LLPS or if they were crystalline formed from supersaturation during dissolution. As seen in Figure 2, WAXS confirms that the nanoparticles were amorphous with only crystalline peaks from the FaSSIF matrix being detected in the sample. No crystalline peaks from the niclosamide anhydrate or niclosamide Ha (hydrate form A) were detected. Importantly, niclosamide is characterized as a poor glass former, which means that it has a high tendency for recrystallization [35]. Niclosamide tends to recrystallize by forming strong hydrophobic π-π interactions [36]. Consequently, Figure 1 and Figure 2 demonstrate that niclosamide can avoid recrystallization by generating these amorphous nanoparticles that can act as reservoirs to increase its oral bioavailability. This formulation strategy can facilitate the development of amorphous solid dispersions containing other poor glass formers. However, these efforts cannot be achieved if the ASD is not effectively translated into a finished dosage form that can be administered to patients. As such, the next challenge is to prevent the recrystallization of niclosamide in the ASD when exposed to acidic conditions that occurs as the dosage form transits through the stomach.

### 3.2. Niclosamide ASD Dissolution Performance and Stability Were Impaired When Formulated as an Enteric Capsule Formulation

**Figure 3:**
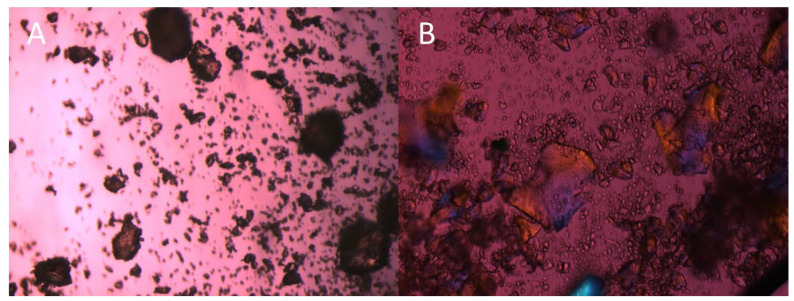
(**A**) Polarized light microscopy of ASD granules (10×); (**B**) same granules 5 min after receiving drops of 0.1 N HCl (20×). The first signs of recrystallizations were observed in less than 1 min.

We previously reported that niclosamide ASD undergoes recrystallization when tested in the pH-shift dissolution test [26]. Figure 3 shows how the ASD granules quickly recrystallized after being wetted by 0.1 N HCl, with the first signs of crystallization taking place after just 1 min. It was concluded that an enteric dosage form was required for a viable pharmaceutical product. This reduction in niclosamide’s solubility in acidic conditions was reported by Yu et al. (2021) when testing niclosamide–montmorillonite hybrid-based formulations [37]. Niclosamide is a weak acid and has an ionizable phenolic -OH group, with reported pKa values ranging from 5.6 to 7.2 [38,39,40,41]. Because of this, the solubility of niclosamide increases at higher pH values and its LogD decreases [38,42]. Needham (2021) experimentally increased niclosamide’s concentration from 2.53 µM at pH 3.66 to 703.5 µM at pH 9.63. In addition to these solubility and solid-state challenges, it was reported that niclosamide undergoes chemical degradation in acidic and in basic media due to the fact of hydrolysis [43,44,45]. It has also been reported that alkaline solutions favor niclosamide’s photooxidation [40]. Niclosamide’s degradation products are toxic to mononuclear cells and neuronal and alveolar cell lines and nontoxic to hepatic cell lines [46].

The niclosamide ASD granules were encapsulated in two different commercially available size 0 capsules: Capsuline^®^ (HPMCP/HPMC) and Eudracap^TM^ enteric (HPMC/Eudragit^®^ polymers) capsules. These enteric capsules contain weakly acidic polymers that release their contents in the intestines, where the polymer is soluble and the capsule shell dissolves to release its payload [47]. In addition, it has been reported that PVP-VA gelling, which is contained in the ASD composition, hinders the disintegration of the dosage forms and their drug release. Gelling can be delayed by adding inorganic salts into the formulation [23]. Therefore, sodium bicarbonate was added for two reasons. First, the bicarbonate ion is a kosmotrope and delays the polymer gelling, and second, it increases the microenvironmental pH inside the formulation to help in the dissolution of the acidic enteric polymer capsule shell [23,48,49,50,51]. In addition, sodium starch glycolate was used as the disintegrant in the capsule formulations.

**Figure 4:**
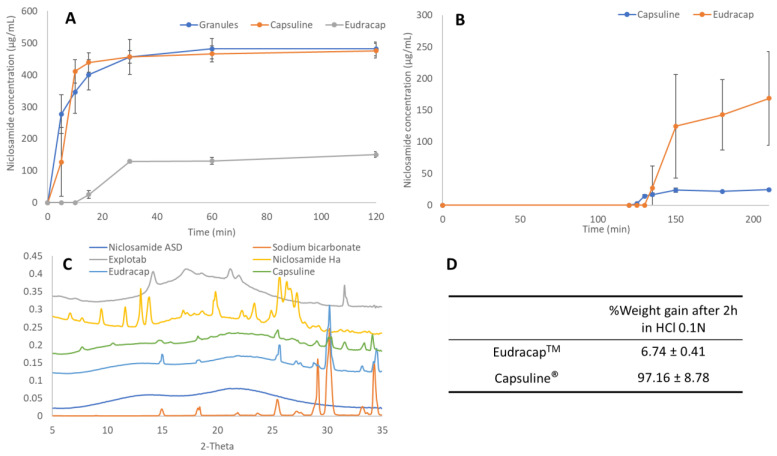
(**A**) Dissolution profile of niclosamide ASD granules (280 mg), Capsuline, and Eudracap capsules (400 mg of 25% sodium bicarbonate, 5% Explotab^®^, and 70% ASD) (n = 3); (**B**) pH-shift dissolution test of the Capsuline and Eudracap capsule formulation; (**C**) WAXS data of filling material contained in the Capsuline and Eudracap capsules after exposure for 2 h in 0.1 N HCl; (**D**) acid uptake test of the capsules after 2 h in 0.1 N HCl.

The dissolution tests in FaSSIF medium were conducted to determine the effect of encapsulation on the performance of the niclosamide ASD granules (Figure 4A). The results confirmed niclosamide concentrations of 476.0 ± 22.9 µg/mL and 150. 7 ± 9.4 µg/mL after 2 h of dissolution for the granules filled into Capsuline^®^ and Eudracap^TM^ capsules, respectively. The Eudracaps^TM^ took longer to dissolve, and this appeared visually to have caused the polymer gelling that resulted in a nondisintegrating ASD plug. For comparison, the extruded and milled granules without additional excipients reached niclosamide concentrations of 481.7 ± 22.2 µg/mL after 2 h of dissolution in FaSSIF. Subsequently, enteric capsules containing niclosamide ASD were tested using pH-shift dissolution tests. The performance of the Eudracap™ was similar but more variable (168.7 ± 73.8 µg/mL) when compared to its performance in the FaSSIF media alone. Surprisingly, the performance of the Capsuline^®^ was greatly reduced to 24.5 ± 2.3 µg/mL due to the premature rupture of the capsule shells in the acidic media (Figure 4B). The 2 h acid phase of this dissolution test was repeated, and the capsules were removed from the dissolution vessel and dried to conduct WAXS. The WAXS revealed that niclosamide in the ASD had crystallized into niclosamide hydrate (Ha) while contained within the Capsuline^®^ capsules. In contrast, Eudracaps^TM^ maintained amorphous niclosamide ASD without crystallization (Figure 4C). An acid uptake test was performed, as shown in Figure 4D. As described in the table, Capsuline^®^ capsules almost doubled their weight due to the rupture of the capsule shell wall. In contrast, the weight gain of the Eudracaps^TM^ was lower after the acid uptake test (Figure 4D). Moreover, the Capsugel^®^ V-Caps^®^ Enteric size 00 capsules were tested and exhibited similar results to the Capsuline^®^ capsules. Based on these results, enteric capsules for use with niclosamide ASD were not considered feasible, thus enteric-coated tablets were developed. Several authors have reported similar results with enteric capsules. Fu et al. (2020) and Al-Tabakha et al. (2014) observed that DRcaps^®^ Enteric (HPMC/gellan gum) from Capsugel ruptured in acidic conditions [52,53]. In addition, Al-Tabakha et al. (2014) also observed capsule rupture with the AR-CAPS^®^ (HPMC/HPMCP) from CapsCanada^®^.

### 3.3. Niclosamide ASD Was Protected by Enteric-Coated Tablet Formulation

To overcome the disadvantages of the enteric capsules, enteric-coated tablets were studied. In general, tablets are a preferred oral dosage form because of their portability, manufacturability, stability, and ease of administration, higher dose precision, and scalability [54,55]. Tablets, as monolithic (i.e., single unit tablet) oral dosage forms, have the advantage of requiring lower amounts of enteric coatings compared to multiparticulate systems, as reported by Missaghi et al. (2010) [56]. In their work related to protecting tablets containing proton pump inhibitors, a 14% weight gain of Acryl-EZE^®^ sufficiently coated the tablets and provided enteric protection. In contrast, multiparticulate systems required weight gains from 26% to even 61.5% of enteric coating [56].

The excipients used in the preparation of niclosamide ASD containing tablets is different from those employed in the capsule formulation. NaCl was incorporated as a kosmotropic salt to facilitate the dissolution of the tablets by reducing PVP-VA gelling [23]. After testing different disintegrants, it was determined that the most suitable disintegrant was croscarmellose sodium. In addition, microcrystalline cellulose was added to increase the hardness and reduce the friability of the tablets to withstand the coating process. The poly (vinyl alcohol)-based coating known as Opadry^®^ II Clear was first used as a seal coating for the tablets using water or ethanol/water mixtures. Opadry^®^ II is a water-soluble and pH-independent coating used for immediate release applications [57]. It was used to reduce the moisture penetration and tablet friability during the subsequent coating steps and to reduce the interactions of the enteric coating with the tablet core. After application of the seal coating, the tablets were enteric coated with Acryl-EZE^®^ 93 A (water) or Opadry^®^ Enteric (ethanol/water).

**Figure 5:**
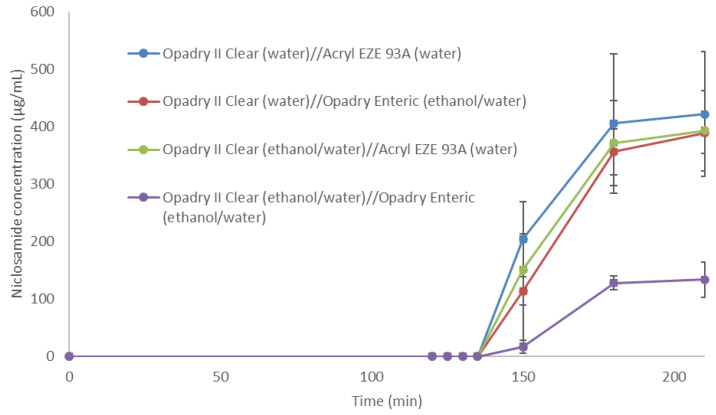
Dissolution profile of the enteric-coated tablets at 10% weight gain.

The enteric-coated tablets containing niclosamide ASD underwent the acid uptake test, and their percentages of weight gain were noticeably lower than those obtained by the enteric capsules (Table 1). Then, the tablets were tested by the dissolution test, as shown in Figure 5. The Opadry II Clear (ethanol/water) overcoated with the Opadry Enteric (ethanol/water) coating noticeably hindered the performance of the ASD formulation. In general, aqueous-based coatings are preferred over organic solvent-based coatings due to the potential toxicity, environmental, and manufacturing safety considerations [56]. In general, the aqueous-based coating formulation achieved better results, maintaining the dissolution performance of niclosamide ASD granules. These tablets were then tested in beagle dogs.

**Figure 6:**
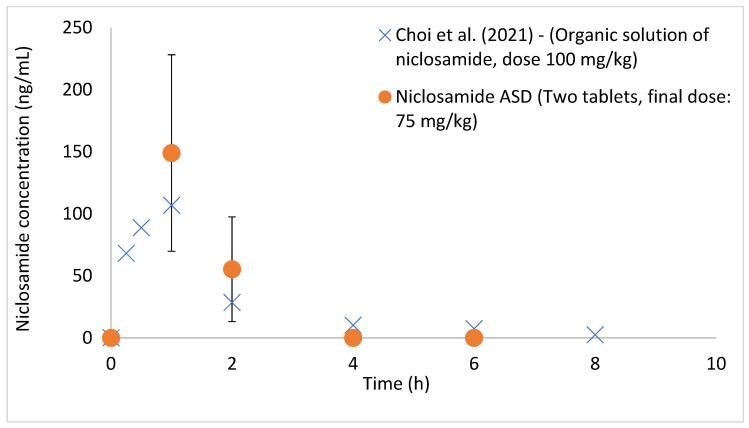
Plasma concentrations of niclosamide in beagle dogs after administering the enteric-coated tablets containing niclosamide ASD (n = 5). Error bars represent standard deviation. The data from Choi et al. (2021) were added to the chart as a comparison [58]. The data were extracted from the publication using the software PlotDigitizer. Reprinted/adapted with permission from Ref. [58]. 2022, Elsevier.

The aqueous-based enteric-coated tablets were administered to fasted beagle dogs, as shown in Figure 6. Plasma concentrations of up to 149 ± 79.2 ng/mL were detected at an administered dose of 75 mg/kg niclosamide (individual results are shown in Figure S3). These plasma concentrations of niclosamide were higher than those previously reported in the literature using solubilized niclosamide administered at a higher dose (100 mg/kg) to beagle dogs [58]. In Choi et al. (2021), niclosamide was dissolved in a mixture containing solvents, polymers, and pH modifiers (DMSO, PEG, NaOH, and saline buffer) [58]. This demonstrates that the enteric-coated tablets can protect niclosamide ASD from the gastric environment, thus preventing its crystallization in acidic conditions.

## 4. Conclusions

We report the development of an enteric-coated tablet dosage form of niclosamide ASD. Niclosamide ASD is an amorphous solid dispersion that generates amorphous nanoparticles during its dissolution in FaSSIF. To maximize niclosamide’s oral bioavailability, the ASD cannot be exposed to acidic conditions due to the fact of API recrystallization and inferior dissolution. To overcome this acidic pH limitation, commercially available enteric capsules were tested. However, their performance was unsatisfactory for a formulation such as niclosamide ASD. Among the tested enteric capsules, acid penetration into the enteric capsules and recrystallization of the API were observed. Additionally, some capsules took too long to disintegrate, leading to polymer gelling and the formation of a nondispersible plug of the ASD within the capsule, negating the solubility advantages of the ASD granules. Therefore, enteric-coated tablets were developed and tested after experiencing the mentioned drawbacks with enteric capsules. The enteric-coated tablets successfully protected the formulation from acid and avoided polymer gelling during the dissolution tests. When tested in a beagle dog model, the enteric-coated tablets achieved increased niclosamide plasma levels when compared to a previously reported oral organic solution of niclosamide. These results will support further in vivo testing of niclosamide ASD.

## Figures and Tables

**Table 1:** Acid uptake test of enteric-coated tablets after exposure for 2 h in 0.1N HCl.

Seal Coating	Enteric Coating	%Weight Gain after 2 h in 0.1 N HCl
Opadry^®^ II Clear (water)	Acryl-EZE^®^ 93A (water)	2.87 ± 0.53
Opadry^®^ II Clear (water)	Opadry^®^ Enteric (ethanol/water)	1.28 ± 0.58
Opadry^®^ II Clear (ethanol/water)	Acryl-EZE^®^ 93A (water)	3.08 ± 0.46
Opadry^®^ II Clear (ethanol/water)	Opadry^®^ Enteric (ethanol/water)	0.94 ± 0.51

## Data Availability

Not applicable.

## Supplementary Materials

The following supporting information can be downloaded at: https://www.mdpi.com/article/10.3390/pharmaceutics14122568/s1, Figure S1: Dissolution profile of niclosamide ASD granules in buffer pH 6.5 and FaSSIF media; Figure S2: (A) Dissolution profile of niclosamide extrudates using different polymers at 40% drug loading (DL) in FaSSIF media. (B) Dissolution profile of niclosamide-PVP-VA extrudates at different DL in FaSSIF media. (C) The dissolution profile of niclosamide-PVP-VA-surfactant extrudates at 20% DL and 5% of surfactants in FaSSIF media; Figure S3: Dispersion of plasma concentrations of niclosamide in Beagle dogs (n = 5) after administering the enteric-coated tablets containing niclosamide ASD; Table S1: Operating temperatures during the extrusion of niclosamide ASD.
