# Supplementary material for: Oral Delivery of Niclosamide as an Amorphous Solid Dispersion That Generates Amorphous Nanoparticles during Dissolution
Source: Pharmaceutics. 2022 Nov 23;14(12):2568. doi: 10.3390/pharmaceutics14122568 (PMC9785291; doi:10.3390/pharmaceutics14122568)
Supplement: Supplementary file 1 [file pharmaceutics-14-02568-s001.zip › pharmaceutics-1979014-supplementary.pdf]

pharmaceutics

MDPI

Supplementary Materials: Oral Delivery of Niclosamide as an Amorphous Solid Dispersion that Generates Amorphous Nanoparticles During Dissolution

Miguel O. Jara, Zachary N. Warnken, Sawittree Sahakijpijarn, Rishi Thakkar, Vineet R. Kulkarni, Dale J. Christensen, John J. Koleng and Robert O. Williams III

Table S1. Operating temperatures during the extrusion of niclosamide ASD.

Zone	Feeder	1	2	3	4	5	6	7	Die
Temperature °C	60	90	140	160	160	160	150	130	115

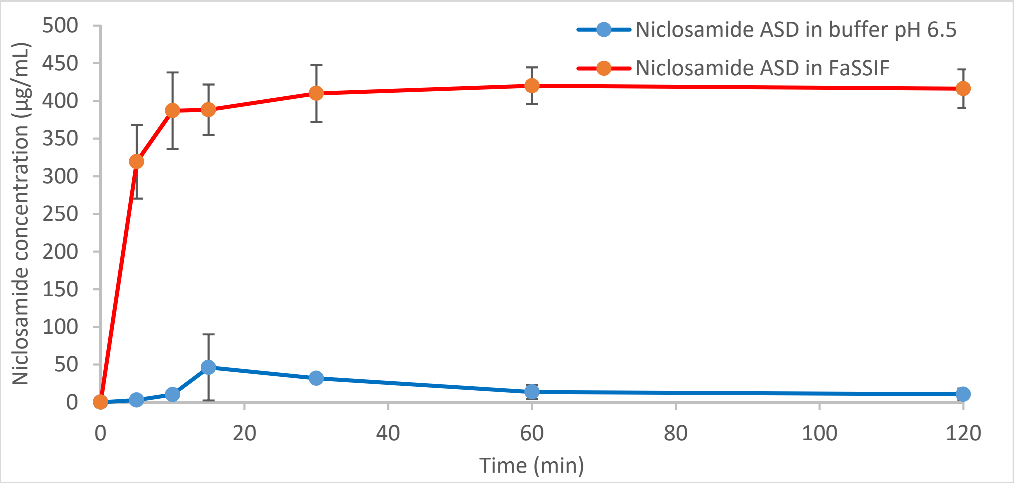

Figure S1. Dissolution profile of niclosamide ASD granules in buffer pH 6.5 and FaSSIF media.

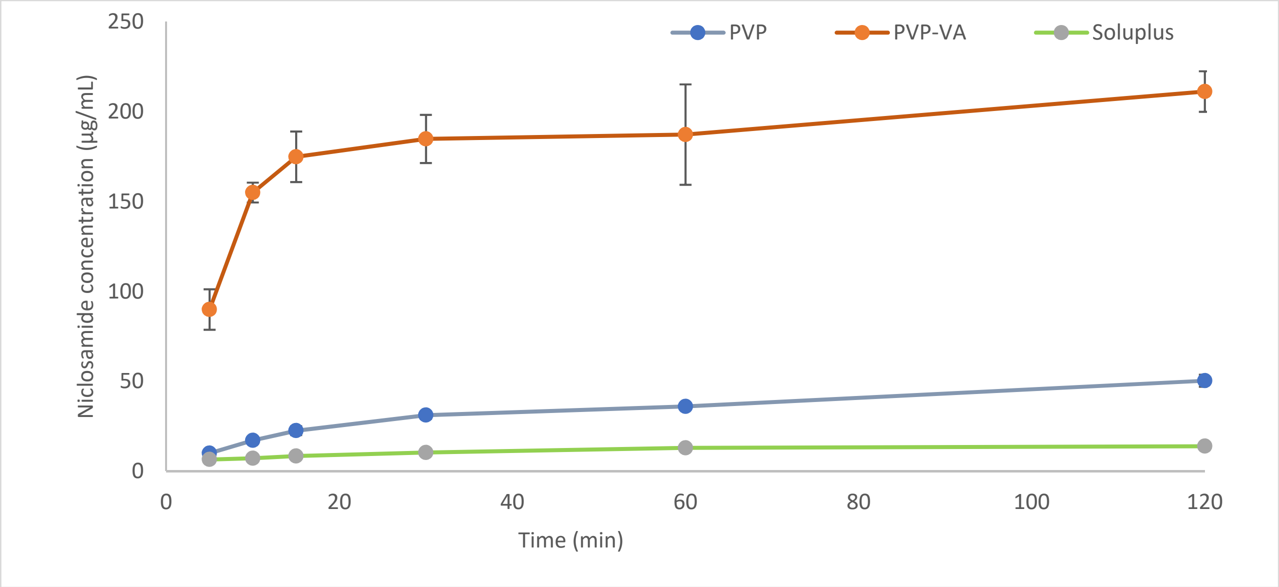

(**A**)

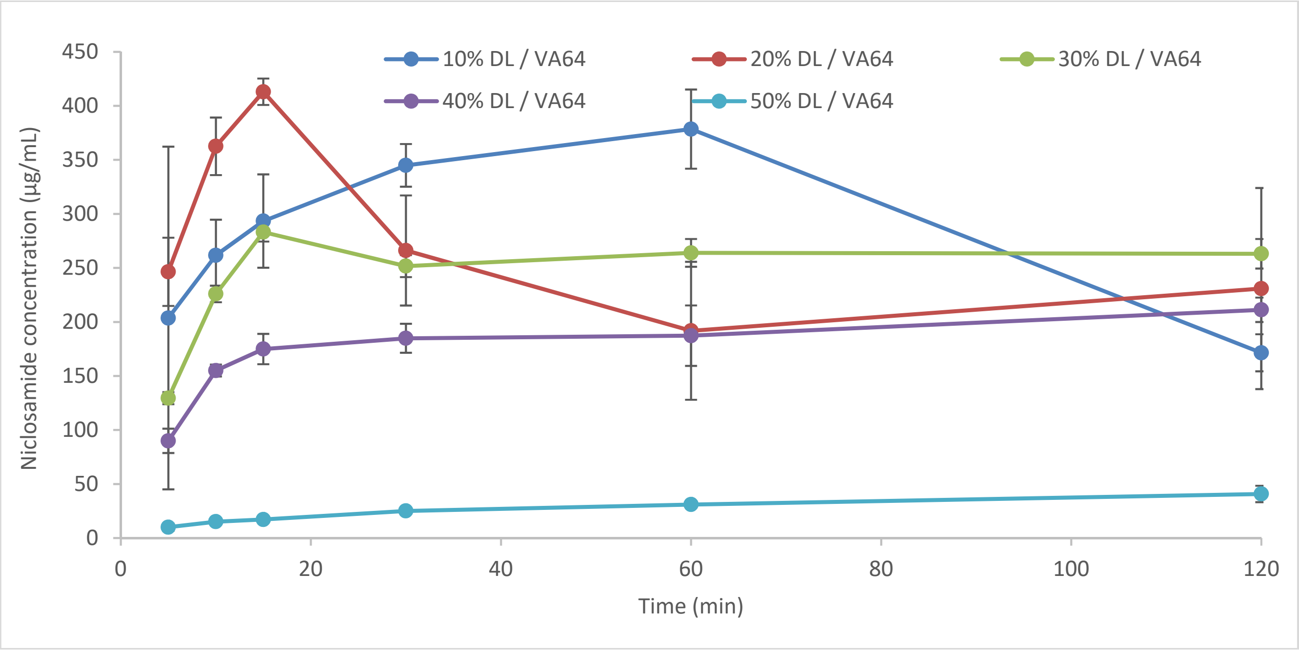

(**B**)

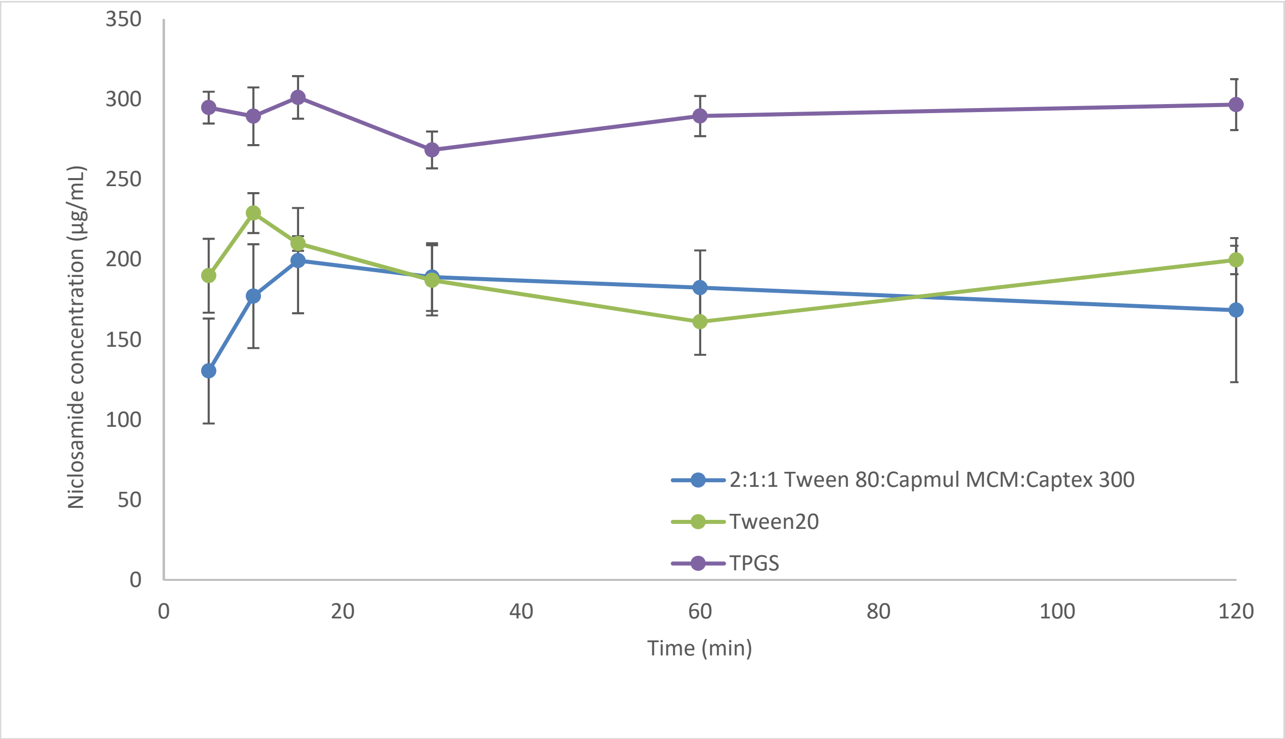

(**C**)

Figure S2. (A) Dissolution profile of niclosamide extrudates using different polymers at 40% drug loading (DL) in FaSSIF media. (B) Dissolution profile of niclosamide-PVP-VA extrudates at different DL in FaSSIF media. (C) The dissolution profile of niclosamide-PVP-VA-surfactant extrudates at 20% DL and 5% of surfactants in FaSSIF media.

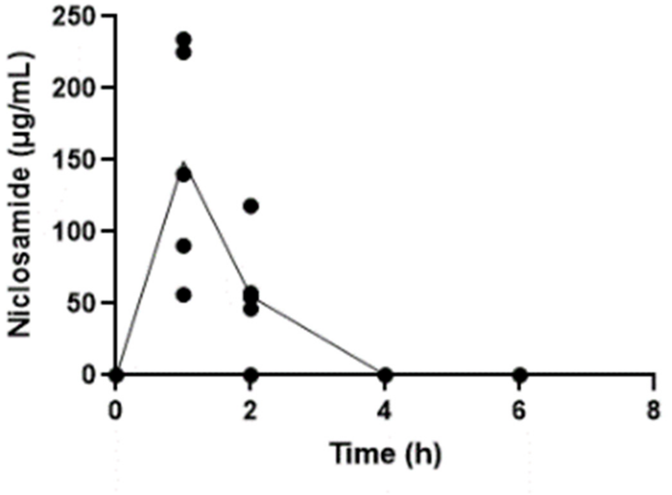

Figure S3. Dispersion of plasma concentrations of niclosamide in Beagle dogs (n=5) after administering the enteric-coated tablets containing niclosamide ASD.
